# A Lightweight Privacy-Enhanced Federated Clustering Algorithm for Edge Computing

**DOI:** 10.3390/s25247544

**Published:** 2025-12-11

**Authors:** Jun Wang, Xianghua Chen, Xing Cheng, Jiantong Zhang, Tao Yu, Kewei Qian

**Affiliations:** 1 College of Computer Science and Technology, Nanjing University of Aeronautics and Astronautics, Nanjing 211106, China; chenxianghua@nuaa.edu.cn; 2 School of Information and Communication Engineering, Beijing Information Science and Technology University, Beijing 102206, China; cheng@bistu.edu.cn; 3 School of Artificial Intelligence, Nankai University, Tianjin 300350, China; 2311180@mail.nankai.edu.cn; 4 CCB Fintech Co., Ltd., Beijing 100094, China; 5 State Grid Shaoxing Power Supply Company, Shaoxing 312000, China

**Keywords:** clustering, edge computing, federated learning, privacy protection, k-means, non-IID

## Abstract

In edge computing scenarios, the data generated by distributed devices is characterized by its dispersion, heterogeneity, and privacy sensitivity, posing significant challenges to federated clustering, including high communication overhead, difficulty in adapting to non-IID data, and significant privacy leakage risks. To address these issues, this paper proposes a privacy-enhanced federated k-means clustering algorithm based on locality-sensitive hashing, aiming to mine latent knowledge from multi-source distributed data while ensuring data privacy protection. The core innovation of this algorithm lies in leveraging the distance sensitivity of clustering pairs, which effectively mitigates the non-IID problem while preserving data privacy and achieves global clustering in just a single communication round, significantly enhancing its practicality in communication-constrained environments. Specifically, the algorithm first evaluates local data dispersion at the client side, dynamically generates cluster cardinality based on dispersion, and obtains initial clustering centers through the k-means algorithm. Subsequently, it employs locality-sensitive hashing to encrypt the center points, uploading only the encrypted clustering information and weight data to the server, thereby achieving privacy protection without relying on a trusted server. On the server side, a secondary weighted k-means clustering is performed in the encrypted space to generate hashed global centers. Experimental results on the MNIST and CIFAR-10 datasets demonstrate that this method maintains robust clustering performance under non-IID data distributions. Most crucially, through a strict single-round client-to-server communication protocol, this approach significantly reduces communication overhead, providing a distributed data mining solution that is efficient, adaptable, and privacy-preserving for resource-constrained edge computing environments.

## 1. Introduction

With the rapid development of the Internet of Things (IoT) and edge computing, a massive number of smart terminals and sensing devices are being integrated into all aspects of production and life at an unprecedented density, giving rise to a new data-centric application paradigm [1]. In this context, data, as a new production factor, has become crucial to its value mining. Clustering analysis, as a classic unsupervised learning method, plays an irreplaceable role in exploring the intrinsic structure of data, identifying user group patterns, and enabling intelligent data management. However, in edge computing environments, data is naturally distributed in a “silo” form across billions of device nodes. Constrained by bandwidth, privacy regulations, and energy consumption [2], the traditional centralized clustering approach of aggregating data to a central server is no longer viable. This new form of data distribution and the new paradigm of computing architecture together constitute a fundamental and pressing research topic in the current field of edge intelligence.

Federated clustering (FC) integrates federated learning and clustering algorithms to mine potential knowledge from multi-source decentralized data under the premise of protecting data privacy [3]. The core advantage of federated learning lies in its ability to achieve knowledge discovery through collaborative model training without directly exchanging raw data, thereby avoiding privacy leakage risks during data transmission [4]. There are many solutions for federated learning clustering. For example, Xu et al. proposed a federated k-means clustering algorithm named FeCA, which adaptively improves local solutions on clients and aggregates them to a central server, demonstrating robustness in various federated scenarios [5]. Holzer et al. proposed a dynamically weighted federated clustering algorithm that optimizes the clustering process by adaptively aggregating cluster assignments and centroids from each data source [6]. Li et al. mitigated the impact of non-IID environments by incorporating prior knowledge from auxiliary models and proximal terms into local objectives, enhancing the robustness and effectiveness of the model [7].

However, existing solutions still exhibit significant limitations: although federated learning (FL) does not directly share raw data, sensitive information may still be leaked through model parameters or gradients uploaded by clients [8]. Attackers can reconstruct original training data through methods such as membership inference [8,9]. In clustering algorithms, the centroids of clusters contain a large amount of data characteristics, which implies significant risks. Additionally, data heterogeneity and communication efficiency are major challenges in federated learning. When the data across clients exhibits non-independent and identically distributed (non-IID) characteristics, the model performance degrades significantly [10]. Data heterogeneity can cause the failure of generic aggregation strategies, potentially leading to global cluster structure confusion (e.g., samples that should belong to the same cluster are split into different clusters, while samples that should belong to different clusters are grouped together). Adapting to low-bandwidth and heterogeneous network environments in edge computing is a notable issue, and frequent model exchanges in distributed architectures introduce substantial communication overhead [11].

To address these issues, this paper innovatively proposes a privacy-enhanced federated learning k-means clustering algorithm based on LSH (locality-sensitive hashing) technology, specifically designed for edge computing scenarios. Compared to existing solutions, the proposed work exhibits distinct differentiation: in contrast to federated k-means and one-time federated clustering algorithms, most existing approaches rely on plaintext-based local centroid aggregation or fixed k-value designs, whereas this work achieves simultaneous resolution of three core challenges—privacy leakage, non-IID data distribution adaptability, and communication efficiency—through hash-space end-to-end clustering and adaptive local k-value adjustment. Regarding LSH application, existing research primarily employs LSH for similarity search or clustering preprocessing in federated learning, failing to achieve end-to-end privacy-preserving aggregation. In contrast, this paper enables the server to perform global clustering directly in the ciphertext space via SimHash-encrypted local centroids, allowing cluster assignment determination for local data without decryption operations.

Specifically, the algorithmic workflow is as follows: The client first evaluates the local data dispersion and generates a scaling factor p, adaptively adjusting the k-value based on p; subsequently, it performs k-means clustering to obtain cluster centroids and data counts, encrypting the centroids using LSH technology; all clients then upload the encrypted centroids and cluster data counts to the server; the server employs weighted k-means to aggregate the hashed centroids, deriving global abstract cluster centroids; clients can determine cluster membership for local data by hashing it via the SimHash algorithm. The core innovations of this paper are summarized as follows: by encrypting centroids with SimHash, conducting all global clustering operations entirely within the hash space, and integrating adaptive k-values with a strict single-round communication design, we achieve synergistic optimization of privacy preservation, non-IID robustness, and communication efficiency.

Our main contributions are as follows:

1. Establishing an end-to-end privacy-preserving clustering pipeline through LSH-based irreversible encryption and hash-space clustering, which neither exposes raw data nor reveals exact centroids, fundamentally circumventing privacy leakage risks.
2. Designing an adaptive local cluster cardinality adjustment mechanism based on data dispersion, enabling the local k-value of each client to align with data distribution complexity, thereby significantly enhancing the algorithm’s robustness to non-IID distributed data.
3. Proposing a strict single-round communication protocol where clients upload encrypted local clustering results only once, and the server performs global aggregation, reducing communication load while maintaining superior distance quality compared to baseline algorithms.
4. Extensive experiments on the MNIST and CIFAR-10 datasets validate that under non-IID settings, the proposed algorithm achieves superior clustering performance over existing solutions, with quantitative analysis further elucidating the impact of LSH techniques and dispersion metrics on algorithmic effectiveness and stability.

The structure of our paper is organized as follows: Section 2 discusses existing federated learning data security issues and data heterogeneity problems; Section 3 elaborates on our algorithm design in detail; Section 4 describes the experimental processes and result analysis conducted to validate our algorithm; and finally, Section 5 presents the conclusion of the paper.

## 2. Related Work

### 2.1. Federated Learning Privacy Preservation Techniques

The advantage of federated learning lies in its localized data storage and training, which avoids the direct transmission of raw data. However, there is still a risk of privacy leakage from information uploaded by clients, such as model parameters, gradients, or cluster centers. For instance,

1. Gradient/Parameter Leakage: Model gradients or parameters uploaded by clients may implicitly contain raw data information. Attackers can reconstruct training data via Gradient Inversion Attacks [12].
2. Membership Inference Attacks: Attackers attempt to infer information about training data (e.g., membership, attributes, or labels) from global or local models [13].
3. Model Extraction Attacks: Attackers eavesdrop on or monitor model parameter updates to extract information about the global model [12].

Current mainstream privacy preservation techniques in federated learning can be categorized as follows:

1. Differential Privacy (DP): By adding carefully designed noise to data or parameters, it masks sensitive information of individual data samples, with its security rigorously proven [14]. However, there exists an inherent trade-off between noise intensity and model performance—excessive noise leads to significant degradation in clustering accuracy.
2. Secure Multi-Party Computation (SMC): Multiple participants collaboratively perform joint computational tasks through encryption without disclosing their private data (add reference). For instance, Chen et al. [15] proposed a novel SMC algorithm, FL-IPFE, to protect local gradients, eliminating the need for a trusted third party while improving FL efficiency and accuracy. Nevertheless, SMC incurs high computational costs in federated learning.
3. Homomorphic Encryption (HE): It supports direct computation on encrypted data, where decrypted results match those obtained from plaintext computations [16]. This technique enables end-to-end privacy protection but suffers from high encryption overhead and computational latency, rendering it particularly unsuitable for real-time tasks such as high-dimensional data clustering.
4. Anonymization Techniques: Strategies like k-anonymity, l-diversity, and t-closeness are employed to remove identifiable information from data before model training, thereby reducing exposure of sensitive raw data. For example, medical data undergoes de-identification locally before participating in federated training, mitigating patient privacy risks [17].

In existing federated clustering tasks, current privacy preservation techniques struggle to balance privacy protection strength, computational overhead, and clustering performance. This research focuses on clustering algorithms, whose essence lies in partitioning datasets into groups (clusters) based on intrinsic data similarity, ensuring high intra-cluster similarity and low inter-cluster similarity. Consequently, clustering tasks only require preserving local similarity to determine cluster assignments, without the need to reconstruct original data. This insight motivates our adoption of LSH technology—which requires no decryption and incurs low overhead—to achieve synergistic optimization of privacy preservation and clustering performance by maintaining local data sensitivity.

### 2.2. Federated Clustering and Non-IID Adaptability Research

Clustering is key to addressing data heterogeneity. Clustering is widely used to mitigate data imbalance by grouping clients with similar data distributions [18] and performing balance optimization within groups or globally. For example, clustering can rely on local data statistical features (e.g., mean, variance) or gradient similarity to measure data distribution similarity.

However, clustering itself faces challenges under data imbalance. Taking the K-FED algorithm [19] as an example, its aggregation performance degrades significantly under imbalanced data compared to balanced scenarios. In non-IID settings, k-means also encounters critical issues: the mismatch in local and global cluster counts (k), as well as variations in k across clients, necessitates effective k-value adjustment strategies.

To address non-IID challenges, existing solutions include the following: globally consistent federated clustering—mapping client data to a shared latent space via Variational Autoencoders (VAEs) for latent-space clustering [20]; Federated Personalized Clustering (FedPAC) [21]—constraining cluster center distributions across clients via regularization terms, allowing personalized clustering models while sharing partial information; Federated GANs—synthesizing minority-class samples to balance client data distributions [22]; dynamic weighted k-means [6]—proposed by Patrick Holzer et al., replacing standard aggregation with reclustering on cluster centers.

Nevertheless, existing methods predominantly rely on fixed local k-values, making them ill-suited to adapt to client-specific data distribution variations. Inadequate representation of local features in non-IID data can lead to degraded global clustering performance. This motivates our design of a data dispersion-based adaptive local k-value adjustment mechanism, ensuring that the local cluster cardinality aligns with data distribution complexity, thereby balancing representational capability and generalization.

### 2.3. Research on Federated Learning Applications Based on LSH

LSH (locality-sensitive hashing) technology perfectly meets these requirements and exhibits strong adaptability to distributed systems. LSH is designed for approximate nearest neighbor (ANN) searches in high-dimensional data, where the core idea is to map similar data points into the same “bucket” with high probability via hash functions [23]. Kapralov et al. evaluated the efficiency and robustness of LSH under adversarial queries through six experiments [24]. It exhibits good adaptability to distributed environments.

The hash function family H of LSH must satisfy the following:

1. High probability of collision for similar data:
  If d(x,y)≤R, then $P\left[h\left(x\right)=h\left(y\right)\right]\geq p_{1}$;
2. Low collision probability for dissimilar data: If d(x,y)≥cR(c>1), then $P\left[h\left(x\right)=h\left(y\right)\right]\leq p_{2}\left(p_{1}\gg p_{2}\right)$.

Here, d(x,y) is the distance metric between data points (e.g., Euclidean distance, cosine similarity, etc.).

Taking SimHash [25] as an example, it is a specific implementation of LSH. SimHash is a concrete LSH variant designed for rapid similarity detection in high-dimensional sparse vectors (e.g., text TF-IDF vectors). Its principle involves projecting high-dimensional vectors into low-dimensional binary hashes via random hyperplanes, ensuring that similar vectors exhibit small Hamming distances in their hash values (Algorithm 1).
**Algorithm 1** SimHash algorithm steps**Input:** High-dimensional vector x∈ Rd.**Output:** Fixed-length binary hash value (e.g., 64-bit)  Steps:1:     Generate random hyperplanes:     Randomly generate d-dimensional Gaussian-distributed vectors r1, r2, …, r64 ∈ Rd, where each $r_{i}$ corresponds to one bit of the hash value.2:     Compute projection signs:     For each $r_{i}$, compute the projection of x: $s_{i}$ = sign($r_{i}$·x). If $r_{i}$· x ≥ 0, set $s_{i}$ = 1; otherwise, $s_{i}$ = 0.3:     Combine hash values:     Concatenate all $s_{i}$ into a binary hash: h(x) = $s_{1}$$s_{2}$, …, $s_{64}$4:     Similarity measurement:     For two hash values h(x) and h(y), compute the Hamming distance (number of differing bits):     Hamming(h(x), h(y)) ≈ 1 − cos($\theta_{xy}$), where $\theta_{xy}$ is the angle between vectors x and y.

SimHash exhibits strong locality-sensitive properties:

If cos(x,y) ≈ 1, then Hamming(h(x), h(y)) ≈ 0.

If cos(x,y) ≈ 0, then Hamming(h(x), h(y)) ≈ 0.5.

Currently, the application of LSH technology in federated learning primarily focuses on the following scenarios:

1. Client Clustering and Personalized Learning: LSH is used to identify clients with similar data distributions or model parameters, enabling local model aggregation within each group to alleviate global model convergence challenges [26]. For example, Rakotomamonjy et al. utilized LSH in personalized federated learning to rapidly identify model parameters of similar clients, aggregating updates only from clients within the same group to reduce communication rounds [27]. Yang et al. proposed the OFCHP framework, employing LSH to efficiently identify categories of similar clients [28].
2. Federated Recommendation Systems: Clients use LSH to map user embedding parameters into hash values, which are then perturbed via local differential privacy (LDP) to prevent raw data leakage. For instance, the FRecLSH algorithm uploads hashed user features to preserve privacy while maintaining recommendation efficacy [29].
3. Feature Space Alignment: In vertical federated learning, LSH aligns feature spaces across different clients. For example, hash functions map heterogeneous features into a shared embedding space to enable cross-client collaborative training [30].
4. Communication Efficiency Optimization: Applying hash mapping to model parameters leverages LSH’s dimensionality reduction and fast retrieval properties to reduce transmitted data volume and enhance communication efficiency [26]. Dai H. et al. employed LSH for rapid mapping and similarity retrieval of federated aggregation model parameters, protecting user privacy and shortening training time [31].
5. Secure Aggregation: In distributed gradient-boosted decision tree (GBDT) frameworks like SeFB, LSH securely constructs tree structures by collecting similarity information of instances without exposing participants’ raw data [32].

It is noteworthy that existing studies have not yet applied LSH technology to end-to-end privacy preservation and aggregation in federated clustering, failing to fully exploit its locality sensitivity and irreversibility to achieve aggregation in the hash space and cluster assignment determination without decryption. This motivates our work to utilize SimHash for encrypting local cluster centroids, enabling the server to perform aggregation entirely in the hash space, while clients can determine local data cluster membership without decryption.

### 2.4. A Typical Federated k-Means Algorithm: FeCA

The FeCA [5] algorithm leverages the structured characteristics of local solutions in k-means clustering within a federated learning environment. It refines local solutions on individual clients and then aggregates them at the central server to reconstruct the global solution (Algorithm 2).
**Algorithm 2** FeCA**Input:** Distributed dataset $D=\left\{D_{1},D_{2},\ldots ,D_{m}\right\}$, where $D_{i}$ is the data on the i-th client. Number of clusters k.**Output:** Global cluster centers C* = $c_{1}$, $c_{2}$, …, $c_{k}$** Steps:**** for** each client = 1, …, M **do**    $C^{\left(m\right)},D^{\left(m\right)}<$— ClientUpdate(k)    //Local execution of k-means with Cluster Center Optimization.    $R^{\left(m\right)}<$—RadiusAssign($C^{\left(m\right)},D^{\left(m\right)}$ )   //Assign a specific radius to each centroid. **end for**    C< — $U_{m=1}^{M}C^{m}$    R< — $U_{m=1}^{M}R^{m}$    $C^{*}<$ —ServerAggregation(k,C,R)    return $C^{*}$

Xu et al. [5] posit that the relationship between local clustering centroids on clients and the global true clustering centroids can be categorized into two scenarios:

Case 1: (one-fit-many association): centroid ci is associated with s (s > 1) true centers $\left\{c_{j1}^{*},\ldots ,c_{js}^{*}\right\}$.

Case 2: (one/many-fit-one association): t (t≥ 1) centroids $\left\{c_{i1},\ldots ,c_{it}\right\}$ are all associated with one true center $c_{j}^{*}$.

The ClientUpdate step determines the type of local centroids based on the following objective function, removes centroids of the one-to-many type, and merges centroids in the many-to-one scenario.

$$G_{i}^{\left(m\right)}=\sum_{x\in D_{i}^{\left(m\right)}}{\left\|x-c_{i}^{\left(m\right)}\right\|}_{2}^{2}$$

The RadiusAssign step assigns a specific radius to each centroid to prepare for server-side aggregation. This step has two algorithmic variants:

1. Theoretical Variant: This assumes the data follows a random spherical model, determines the radius for each clustering center via maximum distance, and filters out global centers.
2. Empirical Variant: This is more suitable for real-world scenarios. It assumes only the need to exclude erroneous one-to-many clustering centers and assigns a unique radius to each remaining center.

In the ServerAggregation(k, C, R) step, the server selects the top-k centroids that cover the maximum number of other center points as candidate center sets and then calculates the average of centroids within each set to obtain the true global clustering centers.

The FeCA algorithm enhances the robustness of federated clustering through local solution optimization and radius-assisted aggregation, but it still exhibits significant limitations: it relies on plaintext transmission and aggregation of local cluster centers, posing privacy leakage risks, and it employs a fixed local k-value design, offering limited adaptability to non-IID data. This motivates our proposed approach of hash-encrypted aggregation and adaptive k-value algorithms to improve both non-IID adaptability and privacy protection.

## 3. Method

### 3.1. Problem Definition and Assumptions

Assume an edge computing scenario with one central server and M honest clients. All clients strictly adhere to the protocol for local computation and data upload, without actively disclosing their private data or fabricating information.

#### 3.1.1. Local Data Definition

The global dataset *D* is distributed across clients, where $D^{g}$ denotes the dataset of the g-thclient containing $n^{g}$ data samples, each represented as a d-dimensional vector, $\sum n^{g}=n$. Let $B^{g}$ denote the set of cluster centers for the g-th client, with kg clusters generated locally based on the data dispersion metric, $B^{g}=$ {$B_{1}^{g},B_{2}^{g},\ldots ,B_{k^{g}}^{g}$}. For each cluster $n^{g}=\sum n_{i}^{g}$, the sum of all $n_{i}^{g}$ in a client corresponds to the total number of data samples on that client. $W^{g}=\left\{w1,w2,\ldots ,w_{k^{g}}\right\}\;$ represents the weight set of the g-th client.

#### 3.1.2. Core Objective

Without directly transmitting raw data, we aim to achieve the global clustering task through collaboration between clients and the server, such that similar data samples are assigned to the same cluster, and clients can accurately determine the cluster assignments of their local data.

#### 3.1.3. Data Characteristic Assumption (Non-IID Definition)

This work focuses on typical horizontal non-IID data distributions in edge computing, specifically defined as follows: significant disparities exist in the class distributions, data features, or statistical properties of local datasets across clients. For example,

1. Class distribution imbalance: Some clients predominantly contain specific classes of data samples, while other classes are scarce.
2. Feature distribution shift: Notable differences exist in statistical characteristics (e.g., mean, variance) of data across clients, leading to deviations between local and global data distributions.

This assumption aligns with the practical scenario of data “silos” in edge computing and constitutes a primary cause of performance degradation in traditional federated clustering algorithms.

#### 3.1.4. Communication Constraint Assumption

Communication constraints in edge computing are defined as follows: limited bandwidth and transmission delays exist between clients and the server; clients are often resource-constrained devices (e.g., sensors, smart terminals), necessitating minimized communication overhead (including data volume and communication rounds) to avoid excessive energy consumption. Based on this, our algorithm strictly follows a single-round communication design to accommodate edge computing limitations.

#### 3.1.5. Threat Model Assumption

This paper adopts the classic Honest-but-Curious (HBC) threat model in federated learning:

Server: It faithfully executes global aggregation computations according to the protocol, without actively tampering with data or interrupting the protocol, but may attempt to infer clients’ original data features or sensitive information from uploaded encrypted cluster centroids, cluster data counts, etc.

Clients: They honestly perform local computations and data encryption and do not engage in collusion attacks or data fabrication; no direct communication exists between clients, eliminating cross-client data leakage risks.

### 3.2. Algorithm Design

Based on the above studies, we intend to generate the local cluster count on the client side according to data dispersion and then perform k-means clustering to obtain cluster centers and cluster population data. After encrypting the cluster centers using the LSH algorithm, they are transmitted to the server. The server then executes a weighted clustering algorithm to aggregate all cluster centers (Algorithm 3 and Table 1).

1. Server Side:
  Distribute the global cluster count global_k to all clients.
2. Client Side:
  Step 1: Each client generates its local cluster count k based on its local data:
  The local cluster number k is dynamically determined by evaluating data dispersion, ensuring the centroid distribution fully reflects true data characteristics. Dispersion is calculated using the total variance of local data. The generation of the local cluster count k proceeds as follows:
  (1) Dispersion Calculation
    Dispersion calculation formula:
    (1) $$d_{g}=\lambda \frac{v_{g}}{n_{g}}$$
    where
    $d_{g}$ is local data dispersion of the g-th client;
    $v_{g}=trace\left(\sum_{g}\right)$ denotes trace of the local data covariance matrix of the g-th client which reflecting the degree of data distribution dispersion;
    $n_{g}$ is the number of local data samples of the g-th client;
    λ is dispersion coefficient (global hyperparameter) which is used to balance the influence of data scale and variance on dispersion. λ∈[0,1]. When λ=0, the global k-value is used, and the dispersion-based local k-value generation is disabled. When the sample sizes across clients vary significantly, set λ∈[0,0.5] to avoid large-sample clients’ $n_{g}$ excessively suppressing dispersion. When the data feature dimensionality is high or the overall variance is relatively large, set λ∈[0.5,1] to enhance the sensitivity of dispersion to data differences.
  (2) Mapping Rules for Local Cluster Number $local_{k}$:
    $$local_{k}=\left\{\begin{array}{cc} 1 & \mathrm{if}\;d_{g}<1\\ 10*global\_k & \mathrm{if}\;d_{g}>10\times \;global_{k}\\ math.floor(d_{g}) & \mathrm{otherwise} \end{array}\right.$$
  Based on the dispersion $d_{g}$ and the globally preset cluster number $global_{k}$, normalized mapping is achieved through the following piecewise function (ensuring reasonable $local_{k}$ values that align with data characteristics).
  Normalization design: Using 1 as the data concentration threshold and $10\times global_{k}$ as the upper limit of dispersion, the $d_{g}$ values from different clients are normalized to a unified interval for mapping, avoiding extreme $local_{k}$ values due to data distribution differences.
  Example: Assume $global_{k}=3,\;\lambda =1$.
  - Client A: $v_{g}=2.8,n_{g}=4\to d_{g}=2.8/4=0.7$. Since $d_{g}<1,\;local_{k}=1$, indicating concentrated data where a single cluster suffices.
  - Client B: $v_{g}=25.2,n_{g}=4\to d_{g}=25.2/4=6.3$. Since $1<d_{g}<10\times 3=30$, $local_{k}=floor\left(6.3\right)$, indicating moderate data dispersion where the cluster number matches the dispersion level.
  - Client C: $v_{g}=100,n_{g}=3\to d_{g}=100/3\approx 33.3$. Since $d_{g}>10\times 3=30,\;local_{k}=30$, indicating highly dispersed data where the upper limit constrains the cluster number.
  Step 2: The client generates the local centroid set ${\left\{b_{i}\right\}}_{i=1}^{k}$ for its data using the standard k-mean algorithm.
  Step 3: The client iterates through each cluster and generates the centroid weight set ${\left\{\omega_{i}\right\}}_{i=1}^{k}$ based on the cluster sample size.
  Step 4: Process the local centroid set using LSH (locality-sensitive hashing):
  Encrypt the data centroid set with SimHash to obtain the hashed centroid set: ${simhash(b_{i})}_{i=1}^{k}$
  Step 5: Upload to the server:
  The client sends the locally encrypted centroid set ${simhash(b_{i})}_{i=1}^{k}$ and the corresponding weight set ${\omega_{i}}_{i=1}^{k}$ to the server.
3. Server Side:
  The server performs weighted k-means on the globally encrypted centroid set to re-cluster and generate global abstract cluster centers. These global abstract cluster centers are then distributed to all clients.
  The core idea of the server-side global aggregation algorithm is to assign weights to each cluster center based on the sample size of its corresponding local cluster—the larger the local cluster, the greater its influence on the global center, ensuring the aggregation results better align with the global data distribution.
  The mathematical definition of weighted k-means aggregation is as follows:
  Input: The server constructs a global ciphertext space centroid set $B_{all}=\cup_{g=1}^{M}B^{g}$, and its corresponding weight set $\Omega_{all}=\cup_{g=1}^{M}\Omega^{g}$.
  Output: C = $\left\{c_{1},c_{2},...,c_{k}\right\}$.
  Objective Function: $min\sum_{j=1}^{k}\sum_{b\in cluster_{k}}\omega_{b}\cdot {∥b-C_{j}∥}_{2}^{2}$, where $\omega_{b}$ is the weight of ciphertext centroid b (derived from the local cluster size $\Omega_{all}$), and  $∥b-C_{j}{∥}_{2}^{2}$ is the squared Euclidean distance in the ciphertext space.
  Computationally, it is equivalent to creating an expanded dataset where each data point is replicated a number of times proportional to its weight, followed by standard k-means clustering.
  To use the abstract cluster centers, local data can be hashed using the SimHash algorithm, and distances to the global abstract cluster centers are then computed to determine the true cluster assignments.
  The algorithm requires only one-shot client upload after initialization, as follows: The server first distributes the k-value to clients. Clients then upload their centroid sets and weight sets to the server, which computes the global abstract cluster centers. Finally, the server distributes these global abstract cluster centers back to all clients.

Algorithm pseudocode:
**Algorithm 3** Proposed Algorithm**Input:** Distributed dataset $D=\left\{D_{1},D_{2},\ldots ,D_{m}\right\}$, where $D_{g}$ is the data on the g-th client. Number of clusters k.**Output:** Global cluster centers C* = $c_{1}$, $c_{2}$, …, $c_{k}$**  Setps:**1:     The server distributes global_k to each client2:     Local client processing (for the g-th client):        Compute local data discreteness        Generate local local_k value        Obtain local cluster centers via k-means algorithm        Iterate through clusters:        for client in client_list:           for cluster in cluster_list:$$B=B\cup B^{g}$$           Within the aggregated cluster center set B:               Initialize cluster centers by randomly selecting k data points as initial global cluster centers            Execute k-means clustering on the sample set using the initialized global cluster centers to obtain final cluster centers $C:\{c_{1},c_{2},\ldots ,c_{k}\}$

### 3.3. Data Heterogeneity Adaptability Analysis

This algorithm addresses data heterogeneity by employing sum utilization, discrete optimization, and aggregation algorithm optimization to mitigate data heterogeneity issues, thereby enhancing the algorithm’s adaptability to data heterogeneity.

The discrete degree of this algorithm is calculated based on the total variance of local data.

The trace of the covariance matrix, which essentially represents the sum of variances across all features, is also referred to as the total variance. It measures data dispersion from two key perspectives:

1. Intuitive reflection of cumulative independent dispersion across all dimensions: The more dispersed a single feature is, the larger the total variance. The total variance aggregates the dispersion of all dimensions into a scalar value, describing the overall dispersion of data across all dimensions.
2. Equivalence to the average squared Euclidean distance from data points to the mean center: The larger the squared distance, the more dispersed the data, and the larger the total variance.

However, it also has certain limitations. The total variance can only reflect the sum of independent dispersion across dimensions and does not include covariance information between features, making it prone to overlooking correlations between features. To comprehensively measure the dispersion of high-dimensional data, subsequent analysis can also consider combining determinants (generalized variance) and eigenvalues to fully characterize the distribution features of the data.

### 3.4. Privacy Protection Strategy

The privacy protection of this scheme is heuristic, achieved through the irreversibility of LSH. The core of LSH’s irreversibility lies in the information compression and many-to-one mapping inherent in the hash design. Compared to differential privacy (DP), LSH does not require injecting noise, thereby avoiding the direct impact of noise on model performance. However, its limitation is the lack of a quantifiable privacy boundary, preventing explicit definition of “the degree of influence of a single data sample on the output” through an ϵ-value as in DP. The privacy protection strength of this work is substantiated by analyzing the design characteristics of the hash functions and potential attack scenarios.

#### 3.4.1. Core Mechanism of LSH for Privacy Protection

The primary goal of LSH is to address the approximate nearest neighbor search problem for high-dimensional data. Directly computing similarity between high-dimensional data points is computationally expensive. LSH addresses this by designing locality-sensitive hash functions that map high-dimensional data to low-dimensional hash values. Under this design, the probability that “similar original data → same/nearby hash values” is significantly higher than that of “dissimilar original data → same/nearby hash values.”

For example, in random projection hashing, a random hyperplane is generated, and data points are mapped to either side of the hyperplane. Multiple projections yield a binary hash. Here, similar data points are more likely to be projected to the same side, resulting in similar hash values.

The irreversibility of LSH stems from the following:

1. Many-to-one mapping of hash functions. Multiple original data points correspond to the same hash value. There is no unique inverse mapping, making recovery impossible.
2. Active information discarding during mapping. The design principle of LSH hash functions is to preserve similarity, not complete information. During mapping, vast amounts of information irrelevant to similarity are actively discarded. For instance, in random projection hashing, only the side of the random hyperplane is retained, while information such as the distance to the hyperplane and its distribution is discarded.
3. Randomness of mapping. The non-deterministic design of hash functions enhances irreversibility. Most LSH hash functions incorporate random parameters, such as randomly selected hyperplanes or sampled parameters. Even for the same original data, different random parameters result in different hash values.

These characteristics enable LSH to fundamentally block the direct reconstruction path of raw data while improving computational and communication efficiency, thereby preventing the leakage of sensitive information during data transmission.

#### 3.4.2. Analysis of Potential Attack Scenarios and Defense Capabilities

Although the irreversibility of LSH provides foundational privacy protection, it may still face the following potential attacks in federated learning scenarios. This section analyzes LSH’s defense effectiveness in conjunction with the attack principles.

1. Cluster Centroid Reconstruction Attack Based on Multiple Hash Versions
  An attacker (such as an honest-but-curious server) might attempt to collect multiple hash versions of the same cluster centroid generated under different random parameters, or aggregate relevant hash values uploaded by multiple clients, in an attempt to reversely derive approximate features of the local cluster centroid through statistical analysis or probabilistic modeling.
  Defense Effectiveness: It is difficult for this attack to succeed, primarily because
  - The random parameters of LSH are independent, and the hash values of the same centroid under different parameters exhibit no clear correlation, making it impossible to construct an effective feature mapping from multiple hash versions.
  - The information discarded during the mapping process is random and irrecoverable. Even with multiple hash versions, it is infeasible to restore key features unrelated to similarity (e.g., variance, extreme values of data distribution), rendering it challenging to reconstruct a meaningful approximation of the cluster centroid.
2. Membership Inference Attacks
  An attacker attempts to infer whether a specific sample belongs to a client’s local dataset by analyzing the similarity between uploaded hash values and known samples, or deduces sensitive information such as data categories or sample counts of a particular client from the distribution characteristics of hash values in the global clustering results.
  Defense Effectiveness: The success rate of this attack is significantly reduced due to the following:
  - The locality-sensitive property of SimHash only ensures small Hamming distances for similar data, but dissimilar data may still collide with low probability, preventing the attacker from uniquely determining sample membership through hash similarity.
  - Clients only upload encrypted cluster centroid hash values and cluster sample counts, without exposing any individual sample’s hash information, leaving the attacker without direct comparison evidence.
  - Under non-IID data distributions, the hash value distributions of cluster centroids across clients vary considerably, making it difficult to inversely deduce a single client’s local data characteristics from the global hash distribution.
  However, it should be noted that if the attacker possesses a large number of known sample hash values and category information, low-precision membership inference might be achieved through statistical probability. In such scenarios, local differential privacy (LDP) can be incorporated to slightly perturb the hash values, further enhancing defense capabilities.
3. Hash Collision Attacks
  An attacker attempts to construct specific hash values that collide with the target client’s cluster centroid hash values, thereby masquerading as legitimate samples or disrupting global clustering results.
  Defense Effectiveness: This attack is highly infeasible because the hash function family of LSH satisfies the property that dissimilar data have low collision probability [33]. The probability of an attacker constructing a hash value that collides with the target hash value is quite low, and after collision, it can only affect local outcomes of global clustering without obtaining any raw data or cluster centroid information.

#### 3.4.3. Irreversibility Verification Experiment for LSH (Reconstruction Challenge)

To validate the irreversibility characteristics of LSH, we designed a reconstruction attack experiment based on SimHash. The results demonstrate that across hash dimensions ranging from 128 to 1024 bits, attackers consistently fail to generate data that fully matches the original cluster centroid hash values (hash matching rate is 0%), and the similarity between reconstructed data and original data remains at a low level (cosine similarity approximately 0.44–0.45, L2 distance approximately 0.049–0.050) (Table 2). The experimental setup is as follows:

1. Data: We generated 1024-dimensional synthetic data, creating 3 distinct cluster centroids. The data was normalized to simulate real-world data feature distributions.
2. SimHash Configuration: We tested 4 different hash bit lengths: 128-bit, 256-bit, 512-bit, and 1024-bit.
3. Attack Methods: Brute-force search and optimization-based attacks.
4. Evaluation Metrics: Cosine Similarity—directional accuracy (0–1, higher is better); L2 Distance—numerical discrepancy (lower is better); Hash Matching Rate—probability of exact matching with target hash; Reconstruction Difficulty Score—comprehensive evaluation of irreversibility.
5. Results: The results of the LSH reconstruction experiment demonstrate the irreversibility of LSH, ensuring that the original data cannot be recovered. Meanwhile, an average cosine similarity of around 0.45 indicates that the global structural features of the data are partially preserved, while fine-grained details are lost, which makes it viable for subsequent clustering.

**Table 2:** Reconstruction experiment results.

Hash Bits	Avg. Cosine Similarity	Avg. L2 Distance	Hash Matching Rate	Compression Ratio
128	0.438	0.050	0%	8.0x
256	0.451	0.049	0%	4.0x
512	0.449	0.049	0%	2.0x
1024	0.453	0.049	0%	1.0x

In summary, the proposed scheme constructs a heuristic privacy protection mechanism targeting raw data and cluster centroids through the irreversibility, information loss characteristics, and locality sensitivity of LSH, effectively defending against mainstream attacks in federated learning scenarios (e.g., cluster centroid reconstruction, membership inference). For high-privacy requirement scenarios, it can be further combined with differential privacy, homomorphic encryption, and other technologies to build a multi-layered privacy protection framework.

### 3.5. Hash Space Clustering Validity Analysis

SimHash space clustering can approximate clustering in the original feature space, primarily due to its order-preserving property for similarity as a Locality-Sensitive Hashing (LSH) method, which aligns well with the core requirements of clustering tasks. The essence of clustering is to partition data into clusters based on similarity rather than reconstructing the absolute distances in the original space. SimHash ensures a high approximation between the cluster structures in the hash space and the original space through the logic of ’distance correlation → consistent cluster membership.’

If the original feature vectors are L2-normalized (with a unit norm), there is a one-to-one correspondence between the Euclidean distance $d_{E(x,y)}$ and the cosine similarity cos(x,y) in the original space: $d_{E(x,y)}=\sqrt{2(1-\cos(x,y))}$. The similarity order-preserving property of SimHash is as follows:

If cos(x,y) ≈ 1, then Hamming(h(x), h(y)) ≈ 0.

If cos(x,y) ≈ 0, then Hamming(h(x), h(y)) ≈ 0.5.

This leads to the conclusion: in the original space, a small Euclidean distance (high similarity) corresponds to a high cosine similarity, which in turn results in a small Hamming distance (high similarity) in the hash space. Conversely, a large Euclidean distance (low similarity) in the original space corresponds to a low cosine similarity, which results in a large Hamming distance (low similarity) in the hash space. This "distance-similarity" transmission relationship allows the similarity judgment in the hash space to be effectively equivalent to that in the original space.

For any cluster C in the original space, the Euclidean distances between its internal vectors are all smaller than those with vectors outside the cluster (the core definition of a cluster). Based on the aforementioned distance correlation, the Hamming distances between the internal vectors of cluster C in the hash space will also be smaller than those with vectors outside the cluster. This means that in the hash space, the vectors originally within cluster C will still be grouped into the same cluster due to their small Hamming distances, while vectors originally outside the cluster will still be grouped into different clusters due to their large Hamming distances, achieving a high probability of consistent cluster membership.

### 3.6. Computational Overhead

#### 3.6.1. Local Client Computational Overhead

1. **Determination of Dispersion and local k:** This computation is relatively lightweight, with the primary bottleneck being the calculation of total variance $v_{g}$. The total variance requires computing the trace of the local data covariance matrix $\sum_{g}$, which involves first calculating the mean of each feature and then computing the sum of squared deviations of each sample from the mean. The time complexity is $O(d\times n_{g})$, requiring a single pass through all samples ($n_{g}$) and all dimensions (d) to calculate the mean and sum of squares. Dispersion is calculated using the formula  $d_{g}=\lambda \frac{v_{g}}{n_{g}}$ and then mapped to local k using a piecewise function. These are constant-time operations with a time complexity of O(1)).
2. **K-Means Clustering Algorithm:** This section implements the standard K-Means clustering algorithm.
  The time complexity of K-Means is $O(I\times n_{g}\times k\times d)$, where:
  - *I*: convergence iterations
  - $n_{g}$: total data points
  - *k*: clusters number (local_k)
  - *d*: dimensionality
3. **SimHash Mapping of Cluster Centers:** The local_k centroids (each a *d*-dimensional vector $b_{i}$) obtained from the previous step are individually processed using SimHash. The computational complexity of SimHash is linear: O(local_k×M×D), where:
  - *M*: dimension after hashing
  - *D*: original dimension
  This makes SimHash highly scalable in distributed environments. SimHash was designed to enhance computational efficiency.

#### 3.6.2. Server Computational Overhead

On the server side, weighted replication + standard K-Means is used to implement an equivalent weighted K-Means algorithm, with a time complexity of O(I×N×global_k×D), where:

- *I*: number of iterations required for convergence
- *N*: total number of data points
- *D*: dimensionality of each data point after hashing

## 4. Experiments and Results

### 4.1. Experimental Settings

The experimental data uses the MNIST dataset [34] and the CIFAR-10 dataset [35].

#### 4.1.1. MNIST Datasets

The MNIST dataset contains 60,000 training images and 10,000 test images. Each image is a 28 × 28-pixel grayscale representation of handwritten digits from 0 to 9. Every image is associated with a corresponding label that identifies which digit (0–9) the image represents. The non-IID setting is as follows: First, the dataset is sorted by digit labels and then partitioned into 200 data shards, each containing 300 samples. In the system, there are 100 clients, each of which is randomly assigned 1–5 shards (with possible repetition), resulting in 300–1500 samples per client. Most clients are allocated samples from 1 to 5 digit classes.

#### 4.1.2. CIFAR-10 Datasets

This dataset is a widely used benchmark in the field of computer vision and machine learning. It consists of 60,000 32 × 32 color images divided into 10 categories, with 6000 images per class. Compared to the MNIST dataset, CIFAR-10 covers more complex real-world scenarios, such as animals, airplanes, and cars. The data dimension is 32×32×3. The high dimensionality can lead to the failure of distance calculations, so feature extraction and dimensionality reduction should be performed on the dataset first. The feature extraction process utilizes a pre-trained CNN for feature extraction, with the model being MobileNetV2, which outputs feature vectors of 1280 dimensions. These features are then reduced to 100 dimensions using Principal Component Analysis (PCA). A total of 50 clients are set up, with each client allocated an average of 1000 samples. For the IID setting, the dataset is shuffled, and then 1000 samples are assigned to each client. For the non-IID setting, the dataset is first sorted by labels and then divided into 100 data shards, with each shard containing 500 samples. Each client is randomly allocated 1–5 shards (with replacement allowed), resulting in 500–2500 samples per client. Consequently, most clients receive data samples from 1 to 5 distinct classes.

#### 4.1.3. Parameter Settings

- Data Distribution Parameters:
  (1) Total number of clients: [100, 50];
  (2) Client sampling ratio: [0.1–1];
  (3) IID data setting: 0 (No), 1 (Yes);
  (4) Maximum Communication Rounds: 50.
- Dispersion-related parameters: Dispersion coefficient λ: [0, 1], where 0 indicates using the global k-value without enabling localized k-value generation based on dispersion.
- SimHash-related parameters:
  (1) Whether to use SimHash algorithm for data encryption: 0 (No), 1 (Yes);
  (2) Hash dimension [128, 256, 512, 1024, 2048].

#### 4.1.4. Baseline Algorithms

1. Classical Multi-Round Algorithms
  FedAvg [3]: As the most fundamental algorithm in federated learning, it conducts training through multi-round iterative model averaging. This algorithm is introduced to enable comparative analysis of the trade-offs between performance and communication costs with multi-round approaches.
2. Advanced Single-Round Algorithms
  (a) FeCA [5]: This algorithm leverages the structured characteristics of local solutions in k-means clustering within a federated environment. It refines local solutions on each client and subsequently aggregates them on the central server to recover the global solution.
  (b) K-FED [19]: This algorithm posits that the heterogeneity in each client’s data distribution (where each client typically contains only a few global categories of data) contributes to revealing the global clustering structure. K-FED employs a farthest-distance heuristic search to optimize the global cluster centers C.

#### 4.1.5. Evaluation Metrics

Evaluation Metrics: NMI and ARI are adopted as the evaluation metrics.

NMI (Normalized Mutual Information): This is used to measure the consistency between two clustering results (or between clustering results and true labels). It is normalized based on Mutual Information (MI) from information theory. The value ranges are [0, 1], where a higher value indicates better alignment between the clustering results and true labels.

ARI (Adjusted Rand Index): This is used to evaluate the similarity between clustering results and true labels. It measures statistical consistency based on whether sample pairs are grouped into the same cluster, adjusted for “random assignments.” The value ranges are [−1, 1], where a higher value indicates better clustering performance.

### 4.2. Experimental Results

To validate the effectiveness, FeCA, K-FED, and FedAvg were selected as the baseline.

#### 4.2.1. Comparison Between the Proposed Method and Baseline Algorithms

To ensure statistical reliability, each algorithm in this experiment was independently executed 5 times under each parameter configuration, with different random seeds used for client sampling each time. Performance metrics (NMI and ARI) were recorded during the experiments. The final results are reported as mean ± standard deviation, where the mean reflects the average performance level, and the standard deviation indicates the stability of the algorithm’s performance. In this section, the proposed algorithm is configured with a hash dimension of 1024 and a dispersion coefficient of 0.4.

**Figure 1:**
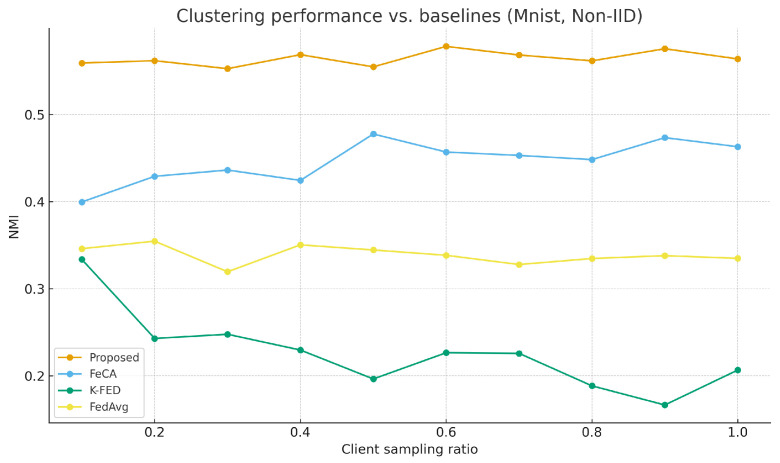
Proposed method vs. Baseline on MNIST (non-IID).

**Figure 2:**
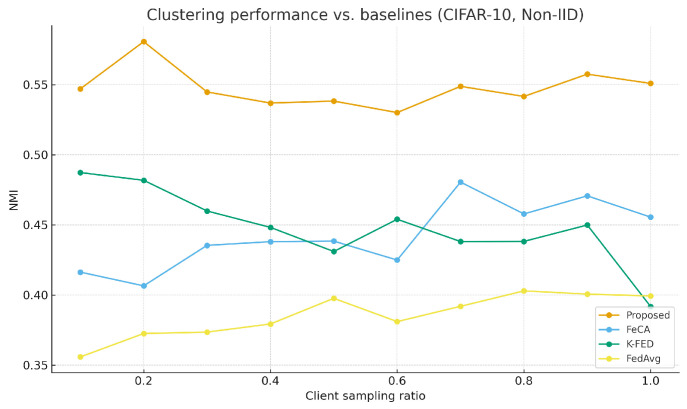
Proposed method vs. Baseline on CIFAR-10 (non-IID).

**Figure 3:**
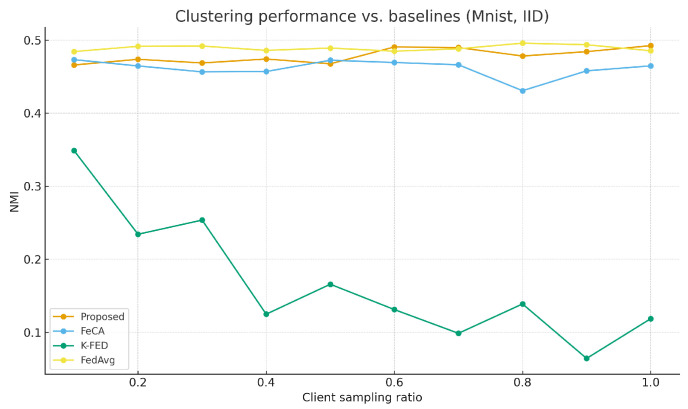
Proposed method vs. Baseline on MNIST (IID).

**Figure 4:**
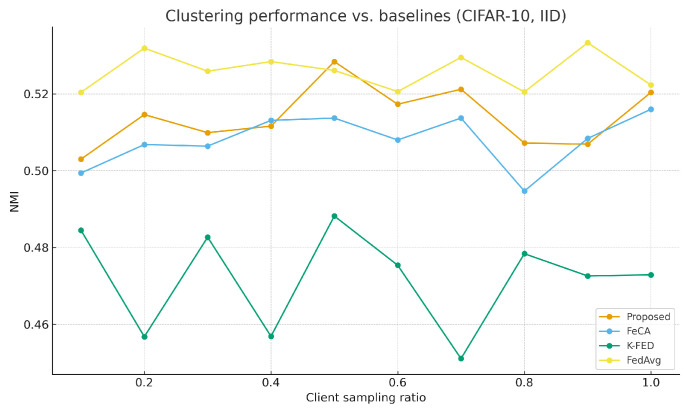
Proposed method vs. Baseline on CIFAR-10 (IID).

As shown in Table 3, the proposed algorithm demonstrates significant advantages under non-IID data settings. Figure 1 and Figure 2 show the performance comparison between our algorithm and the baseline algorithm on the MNIST and CIFAR-10 datasets under non-IID conditions. Figure 3 and Figure 4 show the performance comparison between our algorithm and the baseline algorithm on the MNIST and CIFAR-10 datasets under IID conditions. Compared to single-round algorithms such as FeCA and K-FED, our method achieves notable improvements in both NMI and ARI metrics under non-IID conditions: NMI consistently remains in the 0.53–0.58 range, and ARI reaches 0.41–0.48, representing an approximate 20–40% enhancement over the baseline algorithms. This superiority stems from the algorithm’s effective extraction of heterogeneous data features from clients and improvements in the aggregation strategy. When the client sampling rate varies, our method exhibits excellent stability, with standard deviations of all metrics maintained at low levels (mostly below 0.05), confirming its adaptability to dynamic client participation in federated environments.

In terms of communication efficiency, our algorithm, being a single-round approach, demonstrates a clear advantage. Compared to FedAvg, which requires multiple rounds of iteration, our method reduces the communication rounds from 16 to 50 (the maximum communication rounds in this experiment were set to 50) to just one round, while maintaining comparable or even superior clustering performance. This improvement in communication efficiency is particularly valuable in bandwidth-constrained scenarios such as edge computing. While FedAvg can achieve clustering quality comparable to our method under IID settings, the number of communication rounds required for convergence fluctuates significantly with the client sampling rate. Moreover, under non-IID conditions, FedAvg’s performance degrades substantially, failing to converge within the maximum communication rounds. This highlights the practicality and robustness of our algorithm in real-world heterogeneous data scenarios.

It is particularly noteworthy that all baseline algorithms utilized pre-trained feature extraction on the CIFAR-10 dataset, which partly explains the relatively improved results of FeCA and FedAvg on CIFAR-10 compared to the MNIST dataset. Despite such comparative conditions, our algorithm maintains a leading advantage under non-IID settings, while performing comparably to the baseline algorithms under IID settings. This finding is significant: under a fair comparison environment (i.e., without additional feature extraction), the advantages of our algorithm are expected to be more pronounced, indirectly demonstrating the superiority of its design.

In summary, our algorithm achieves a better balance between communication efficiency and clustering performance, providing an effective solution for clustering tasks in federated learning.

#### 4.2.2. Impact of LSH Mapping Dimensions on Performance

**Figure 5:**
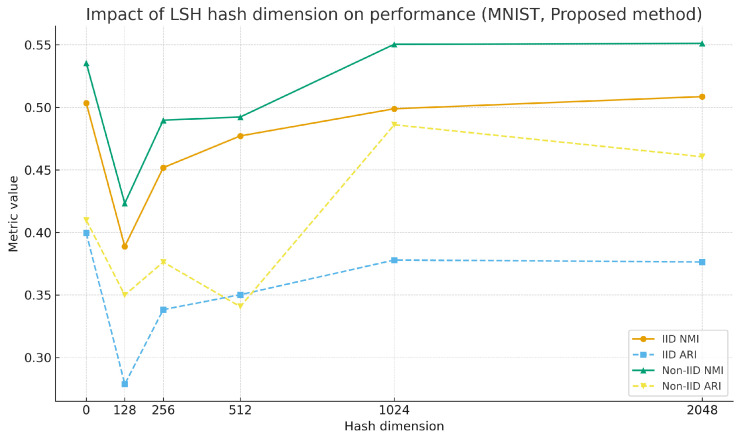
Impact of LSH mapping dimensions on performance on MNIST.

As shown in Table 4 and Table 5, under different client settings, a consistent trend of performance improvement is observed as the hash dimension increases. For instance, on the MNIST dataset, when the hash dimension is increased from 128 to 1024 dimensions, the NMI metric improves by approximately 28%, and the ARI metric improves by about 36%. Once the hash dimension reaches a certain threshold, the performance growth tends to plateau, and in most cases, 1024 dimensions already approach optimal performance. When the mapping dimension decreases, the overlap in low-dimensional projections of high-dimensional data increases, leading to a higher hash collision probability [36]. A single hash value then corresponds to an exponentially increasing amount of original data, making reverse inference extremely difficult and relatively strengthening privacy protection, but with a noticeable performance loss. Higher dimensions maintain good privacy while achieving performance close to the no-hashing baseline. Figure 5 shows the variation in the performance of this algorithm under different hash dimensions on the MNIST dataset.

In the non-IID scenario of the CIFAR-10 dataset (50 clients), 256 and 2048 dimensions exhibit exceptional performance, suggesting that in certain specific configurations, a moderate hash dimension may yield the best results. Additionally, the algorithm demonstrates stable performance under both IID and non-IID settings, indicating its strong adaptability to non-IID data distributions.

In summary, the LSH hash dimension has a significant impact on the performance of the proposed algorithm, with a clear positive correlation between dimension and performance. A 1024-dimensional hash achieves the best balance between privacy protection and clustering performance, ensuring sufficient feature discriminability while controlling computational complexity. It is the recommended default parameter configuration for this algorithm.

#### 4.2.3. Impact of Dispersion on Algorithm Performance

**Figure 6:**
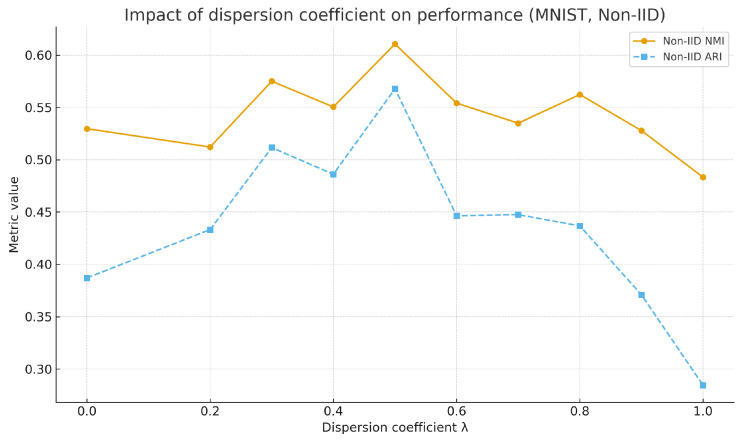
Impact of dispersion on algorithm performance on MNIST.

The algorithm controls the scaling degree of discreteness through a discrete coefficient. As confirmed by the systematic experiments in Table 6 and Table 7, the adaptive discreteness adjustment mechanism demonstrates significant effectiveness in addressing data heterogeneity issues. Compared to traditional methods with fixed k-values, the proposed algorithm dynamically generates cluster cardinalities by evaluating local data discreteness, achieving a performance improvement of 15.3–31.6% in non-IID scenarios. Furthermore, it is observed that there is a clear nonlinear relationship between the discrete coefficient and algorithm performance. On the MNIST dataset, the algorithm performs optimally when the discrete coefficient is in the range of 0.3–0.5, with the NMI metric stably maintained in the high range of 0.553–0.611. The optimal range for the CIFAR-10 dataset is slightly wider (0.3–0.7), but it similarly avoids performance degradation in extreme value regions. This finding validates the superiority of the data-driven adaptive adjustment strategy over fixed-parameter methods. Figure 6 shows the variation in this algorithm’s performance with different discrete coefficients on the MNIST dataset.

## 5. Conclusions

Based on the characteristics of clustering algorithms, this paper innovatively designs a privacy-enhanced asynchronous aggregation federated learning k-means algorithm that incorporates locality-sensitive hashing (LSH) technology. This algorithm can effectively address data heterogeneity and communication efficiency issues in edge computing while preserving privacy. The proposed method first generates a scaling coefficient by evaluating the local data distribution discreteness at each client, based on which a local k-value is determined. Subsequently, k-means clustering is performed to obtain cluster centroids and their corresponding data volumes, followed by LSH-based hashing of the centroids. The hashed cluster centroids and data volume information are then uploaded to the server. The server aggregates the hashed centroids using a weighted k-means algorithm to generate abstract global centroids. Notably, this scheme ensures that clients cannot reconstruct the original centroids but can map their local data to the corresponding clusters via SimHash after hashing, ultimately obtaining local clustering results.

Experiments on the MNIST and CIFAR-10 datasets have validated the effectiveness of the proposed method: it maintains robust clustering performance under non-IID settings while achieving significant improvements in communication efficiency. In summary, this study provides a practical solution to the triple challenges of communication, heterogeneity, and privacy in collaborative learning for edge environments, offering a new technical pathway for achieving efficient, secure, and adaptive edge intelligence data mining.

The LSH-based federated learning clustering scheme proposed in this paper has achieved preliminary results in mitigating data heterogeneity and providing basic privacy protection, but there remains room for optimization: The current privacy protection mechanism relies on the irreversibility of LSH to achieve fundamental security, but it has not yet integrated mechanisms such as differential privacy to establish quantifiable privacy boundaries, which could be further improved in this direction. Furthermore, the current dispersion calculation incorporates two core metrics—total variance and data volume. Future work could attempt to include additional dimensions such as data distribution characteristics, which would better reduce its sensitivity to results and enhance adaptability to more edge device data scenarios.

To address these optimization directions, future work can focus on the following specific paths: First, explore integration patterns between LSH and differential privacy, drawing on dynamic noise injection strategies to construct a dual-layer protection framework. This approach preserves the efficiency of LSH while supplementing it with quantifiable privacy theoretical support. Second, optimize the dispersion calculation model by integrating features such as data skewness coefficient and local density, combined with lightweight learning methods to enhance stability. Third, expand experimental scenarios to validate the scheme’s adaptability in areas such as industrial IoT device operation and maintenance and multi-center collaboration in smart healthcare.

## Figures and Tables

**Table 1:** Symbol explanation.

Symbol	Description
*D*	Global dataset
$D^{g}$	Dataset of the g-th client
$k^{g}$	k-value for the g-th client
$n^{g}$	Number of data samples in the g-th client
$cov^{g}$	Covariance matrix of the g-th client
$di^{g}$	Discreteness measure for the g-th client
$B^{g}$	Cluster center set of the g-th client
$W^{g}$	Weight set of the g-th client

**Table 3:** Performance comparison of the proposed algorithm and baseline algorithms.

Data Sets	Total Clients	Client Sampling Ratio	Proposed Algorithm				FeCA Algorithm				K-FED Algorithm				FedAvg Algorithm					
			IID		Non-IID		IID		Non-IID		IID		Non-IID		IID			Non-IID		
			NMI	ARI	NMI	ARI	NMI	ARI	NMI	ARI	NMI	ARI	NMI	ARI	NMI	ARI	Rounds	NMI	ARI	Rounds
MNIST	100	0.1	0.4659 ± 0.029	0.3339 ± 0.057	0.5592 ± 0.052	0.4356 ± 0.082	0.4732 ± 0.019	0.3380 ± 0.024	0.3996 ± 0.026	0.2845 ± 0.031	0.3489 ± 0.131	0.2288 ± 0.121	0.3337 ± 0.050	0.2060 ± 0.099	0.4843 ± 0.012	0.3589 ± 0.020	33	0.3460 ± 0.032	0.2119 ± 0.035	50
MNIST	100	0.2	0.4737 ± 0.025	0.3316 ± 0.032	0.5619 ± 0.026	0.4668 ± 0.034	0.4646 ± 0.035	0.3404 ± 0.047	0.4291 ± 0.057	0.2948 ± 0.050	0.2342 ± 0.121	0.1208 ± 0.090	0.2429 ± 0.047	0.1624 ± 0.100	0.4915 ± 0.012	0.3598 ± 0.015	19	0.3546 ± 0.017	0.2209 ± 0.023	50
MNIST	100	0.3	0.4687 ± 0.023	0.3410 ± 0.023	0.5527 ± 0.036	0.4234 ± 0.057	0.4565 ± 0.018	0.3261 ± 0.030	0.4363 ± 0.039	0.3054 ± 0.059	0.2536 ± 0.092	0.1236 ± 0.100	0.2477 ± 0.021	0.1097 ± 0.040	0.4919 ± 0.009	0.3665 ± 0.017	37	0.3195 ± 0.013	0.1922 ± 0.031	50
MNIST	100	0.4	0.4741 ± 0.019	0.3442 ± 0.025	0.5688 ± 0.021	0.4448 ± 0.048	0.4570 ± 0.024	0.3370 ± 0.027	0.4244 ± 0.034	0.2956 ± 0.035	0.1248 ± 0.047	0.0167 ± 0.015	0.2296 ± 0.088	0.0897 ± 0.046	0.4859 ± 0.009	0.3582 ± 0.012	35	0.3504 ± 0.015	0.2191 ± 0.018	50
MNIST	100	0.5	0.4675 ± 0.009	0.3382 ± 0.017	0.5548 ± 0.034	0.4240 ± 0.048	0.4724 ± 0.020	0.3414 ± 0.023	0.4777 ± 0.075	0.3635 ± 0.093	0.1658 ± 0.038	0.0368 ± 0.027	0.1964 ± 0.037	0.0571 ± 0.031	0.4891 ± 0.007	0.3584 ± 0.003	28	0.3445 ± 0.024	0.2156 ± 0.037	50
MNIST	100	0.6	0.4906 ± 0.013	0.3746 ± 0.015	0.5783 ± 0.012	0.4579 ± 0.051	0.4693 ± 0.026	0.3458 ± 0.033	0.4570 ± 0.035	0.3442 ± 0.060	0.1311 ± 0.040	0.0306 ± 0.027	0.2265 ± 0.069	0.1086 ± 0.085	0.4848 ± 0.006	0.3596 ± 0.003	34	0.3384 ± 0.012	0.2058 ± 0.020	50
MNIST	100	0.7	0.4898 ± 0.016	0.3706 ± 0.034	0.5684 ± 0.016	0.4434 ± 0.060	0.4662 ± 0.021	0.3423 ± 0.021	0.4531 ± 0.057	0.3545 ± 0.077	0.0986 ± 0.060	0.0146 ± 0.025	0.2258 ± 0.030	0.0815 ± 0.006	0.4881 ± 0.005	0.3667 ± 0.011	19	0.3278 ± 0.019	0.1950 ± 0.027	50
MNIST	100	0.8	0.4782 ± 0.013	0.3491 ± 0.019	0.5617 ± 0.020	0.4357 ± 0.033	0.4307 ± 0.019	0.3061 ± 0.015	0.4483 ± 0.040	0.3322 ± 0.032	0.1388 ± 0.122	0.0490 ± 0.083	0.1885 ± 0.072	0.0709 ± 0.066	0.4958 ± 0.011	0.3771 ± 0.016	27	0.3347 ± 0.018	0.2043 ± 0.027	50
MNIST	100	0.9	0.4842 ± 0.015	0.3635 ± 0.027	0.5756 ± 0.035	0.4805 ± 0.036	0.4579 ± 0.036	0.3372 ± 0.033	0.4735 ± 0.034	0.3484 ± 0.051	0.0642 ± 0.038	0.0037 ± 0.004	0.1665 ± 0.046	0.0427 ± 0.043	0.4936 ± 0.023	0.3726 ± 0.032	41	0.3380 ± 0.018	0.2070 ± 0.025	50
MNIST	100	1	0.4924 ± 0.019	0.3665 ± 0.019	0.5639 ± 0.020	0.4818 ± 0.070	0.4647 ± 0.023	0.3360 ± 0.024	0.4631 ± 0.028	0.3377 ± 0.045	0.1186 ± 0.064	0.0224 ± 0.024	0.2066 ± 0.040	0.0951 ± 0.045	0.4854 ± 0.005	0.3588 ± 0.001	50	0.3349 ± 0.015	0.2049 ± 0.021	50
CIFAR-10	50	0.1	0.5030 ± 0.027	0.3638 ± 0.040	0.5470 ± 0.071	0.4721 ± 0.092	0.4994 ± 0.004	0.3728 ± 0.016	0.4163 ± 0.062	0.2645 ± 0.106	0.4845 ± 0.020	0.3499 ± 0.024	0.4873 ± 0.026	0.3733 ± 0.038	0.5204 ± 0.013	0.3944 ± 0.022	50	0.3558 ± 0.046	0.2197 ± 0.032	50
CIFAR-10	50	0.2	0.5146 ± 0.006	0.3937 ± 0.014	0.5807 ± 0.053	0.4780 ± 0.080	0.5068 ± 0.016	0.3796 ± 0.019	0.4065 ± 0.064	0.2590 ± 0.054	0.4568 ± 0.019	0.3127 ± 0.024	0.4818 ± 0.007	0.3507 ± 0.038	0.5319 ± 0.006	0.4170 ± 0.007	16	0.3725 ± 0.019	0.2434 ± 0.024	50
CIFAR-10	50	0.3	0.5099 ± 0.016	0.3818 ± 0.023	0.5448 ± 0.033	0.4194 ± 0.078	0.5064 ± 0.019	0.3779 ± 0.018	0.4354 ± 0.043	0.3232 ± 0.117	0.4827 ± 0.012	0.3420 ± 0.027	0.4599 ± 0.034	0.3307 ± 0.081	0.5259 ± 0.015	0.4109 ± 0.023	20	0.3735 ± 0.009	0.2285 ± 0.013	50
CIFAR-10	50	0.4	0.5116 ± 0.015	0.3795 ± 0.021	0.5369 ± 0.026	0.4187 ± 0.047	0.5131 ± 0.012	0.3957 ± 0.019	0.4380 ± 0.017	0.3054 ± 0.064	0.4569 ± 0.017	0.3193 ± 0.023	0.4482 ± 0.034	0.2997 ± 0.063	0.5284 ± 0.009	0.4150 ± 0.021	50	0.3793 ± 0.020	0.2432 ± 0.026	50
CIFAR-10	50	0.5	0.5284 ± 0.010	0.4088 ± 0.015	0.5383 ± 0.036	0.4223 ± 0.055	0.5137 ± 0.016	0.3949 ± 0.021	0.4384 ± 0.051	0.2994 ± 0.064	0.4882 ± 0.025	0.3512 ± 0.059	0.4310 ± 0.048	0.2923 ± 0.074	0.5261 ± 0.007	0.4149 ± 0.009	50	0.3976 ± 0.023	0.2717 ± 0.034	50
CIFAR-10	50	0.6	0.5173 ± 0.031	0.3904 ± 0.035	0.5301 ± 0.040	0.4072 ± 0.075	0.5080 ± 0.007	0.3895 ± 0.010	0.4249 ± 0.061	0.2922 ± 0.061	0.4754 ± 0.016	0.3428 ± 0.021	0.4540 ± 0.025	0.3325 ± 0.035	0.5206 ± 0.011	0.4001 ± 0.011	50	0.3810 ± 0.029	0.2395 ± 0.044	50
CIFAR-10	50	0.7	0.5212 ± 0.013	0.3848 ± 0.009	0.5488 ± 0.009	0.4346 ± 0.043	0.5137 ± 0.010	0.3992 ± 0.010	0.4805 ± 0.033	0.3541 ± 0.071	0.4511 ± 0.019	0.3060 ± 0.014	0.4381 ± 0.037	0.3238 ± 0.049	0.5295 ± 0.012	0.4159 ± 0.020	26	0.3919 ± 0.022	0.2555 ± 0.045	50
CIFAR-10	50	0.8	0.5072 ± 0.016	0.3792 ± 0.023	0.5416 ± 0.022	0.4129 ± 0.054	0.4947 ± 0.013	0.3800 ± 0.024	0.4578 ± 0.050	0.3057 ± 0.059	0.4784 ± 0.027	0.3307 ± 0.049	0.4382 ± 0.031	0.3335 ± 0.046	0.5205 ± 0.015	0.4013 ± 0.025	50	0.4029 ± 0.022	0.2640 ± 0.028	50
CIFAR-10	50	0.9	0.5069 ± 0.009	0.3641 ± 0.020	0.5575 ± 0.024	0.4434 ± 0.048	0.5084 ± 0.012	0.3890 ± 0.015	0.4707 ± 0.057	0.3427 ± 0.079	0.4726 ± 0.027	0.3372 ± 0.034	0.4499 ± 0.027	0.3312 ± 0.058	0.5333 ± 0.014	0.4254 ± 0.018	50	0.4006 ± 0.028	0.2660 ± 0.037	50
CIFAR-10	50	1	0.5204 ± 0.002	0.3925 ± 0.015	0.5509 ± 0.023	0.4443 ± 0.047	0.5160 ± 0.015	0.3986 ± 0.021	0.4555 ± 0.062	0.3256 ± 0.085	0.4729 ± 0.026	0.3432 ± 0.032	0.3917 ± 0.086	0.2342 ± 0.136	0.5223 ± 0.016	0.4061 ± 0.022	47	0.3993 ± 0.025	0.2603 ± 0.025	50

**Table 4:** Experimental results on the impact of LSH encryption on algorithm performance on MNIST.

Total Clients	Client Sampling Ratio	Dispersion Coefficient	Hash Dimension	IID		Non-IID	
				NMI	ARI	NMI	ARI
100	0.1	0.4	no hash	0.50347	0.39956	0.53560	0.40984
100	0.1	0.4	128	0.38887	0.27877	0.42331	0.34994
100	0.1	0.4	256	0.45183	0.33833	0.48977	0.37638
100	0.1	0.4	512	0.47719	0.35029	0.49230	0.34073
100	0.1	0.4	1024	0.49890	0.37795	0.55045	0.48612
100	0.1	0.4	2048	0.50861	0.37648	0.55118	0.46056
100	0.5	0.4	no hash	0.49015	0.38036	0.59329	0.53213
100	0.5	0.4	128	0.39151	0.28909	0.49653	0.41342
100	0.5	0.4	256	0.43393	0.30958	0.51918	0.43321
100	0.5	0.4	512	0.48026	0.36850	0.52847	0.41198
100	0.5	0.4	1024	0.49736	0.39615	0.57818	0.45479
100	0.5	0.4	2048	0.49760	0.39478	0.55668	0.45169
100	1	0.4	no hash	0.48622	0.37729	0.55798	0.47063
100	1	0.4	128	0.42086	0.30330	0.45408	0.35483
100	1	0.4	256	0.45829	0.34154	0.50857	0.44490
100	1	0.4	512	0.48247	0.38016	0.55305	0.47906
100	1	0.4	1024	0.50807	0.38749	0.57694	0.49933
100	1	0.4	2048	0.50275	0.41585	0.57123	0.48278

**Table 5:** Experimental results on the impact of LSH encryption on algorithm performance on CIFAR-10.

Total Clients	Client Sampling Ratio	Dispersion Coefficient	Hash Dimension	IID		Non-IID	
				NMI	ARI	NMI	ARI
50	0.1	0.4	no hash	0.53575	0.41009	0.55622	0.45410
50	0.1	0.4	128	0.44043	0.33211	0.41458	0.30337
50	0.1	0.4	256	0.45183	0.33833	0.48977	0.37638
50	0.1	0.4	512	0.47967	0.37563	0.61440	0.60387
50	0.1	0.4	1024	0.49733	0.35148	0.56505	0.50765
50	0.1	0.4	2048	0.51095	0.36885	0.56470	0.57238
50	0.5	0.4	no hash	0.50364	0.37190	0.55301	0.53364
50	0.5	0.4	128	0.41544	0.28841	0.42535	0.28618
50	0.5	0.4	256	0.47741	0.34055	0.52913	0.42075
50	0.5	0.4	512	0.49461	0.36845	0.57021	0.52405
50	0.5	0.4	1024	0.51749	0.37612	0.54859	0.42511
50	0.5	0.4	2048	0.50573	0.38180	0.62095	0.59433
50	1	0.4	no hash	0.53451	0.42206	0.57611	0.48066
50	1	0.4	128	0.43098	0.29746	0.41916	0.28015
50	1	0.4	256	0.49001	0.36862	0.52497	0.42499
50	1	0.4	512	0.49342	0.36196	0.58756	0.50844
50	1	0.4	1024	0.51274	0.36690	0.57791	0.47553
50	1	0.4	2048	0.52294	0.37136	0.55335	0.42793

**Table 6:** Impact of dispersion coefficient on algorithm performance under non-IID settings on MNIST.

Total Clients	Client Sampling Ratio	Dispersion Coefficient	Hash Dimension	NMI	ARI
100	0.1	0 (fixed k-value)	1024	0.52964	0.38695
100	0.1	0.2	1024	0.51212	0.43321
100	0.1	0.3	1024	0.57499	0.51153
100	0.1	0.4	1024	0.55045	0.48612
100	0.1	0.5	1024	0.61058	0.56776
100	0.1	0.6	1024	0.55406	0.44641
100	0.1	0.7	1024	0.53490	0.44748
100	0.8	0.8	1024	0.56225	0.43671
100	0.1	0.9	1024	0.52781	0.37085
100	0.1	1.0	1024	0.54103	0.42364
100	1.0	0 (fixed k-value)	1024	0.48416	0.30397
100	1.0	0.2	1024	0.57039	0.45748
100	1.0	0.3	1024	0.59921	0.57793
100	1.0	0.4	1024	0.58387	0.49105
100	1.0	0.5	1024	0.55358	0.49275
100	1.0	0.6	1024	0.59031	0.43978
100	1.0	0.7	1024	0.55102	0.42057
100	1.0	0.8	1024	0.60150	0.44040
100	1.0	0.9	1024	0.58760	0.39058
100	1.0	1.0	1024	0.57463	0.44178

**Table 7:** Impact of dispersion coefficient on algorithm performance under non-IID settings on CIFAR-10.

Total Clients	Client Sampling Ratio	Dispersion Coefficient	Hash Dimension	NMI	ARI
100	0.1	0 (fixed k-value)	1024	0.49976	0.36753
100	0.1	0.2	1024	0.55258	0.50615
100	0.1	0.3	1024	0.58022	0.65173
100	0.1	0.4	1024	0.49051	0.54088
100	0.1	0.5	1024	0.53991	0.46285
100	0.1	0.6	1024	0.51155	0.39650
100	0.1	0.7	1024	0.65830	0.59588
100	0.8	0.8	1024	0.60112	0.50753
100	0.1	0.9	1024	0.56509	0.58723
100	0.1	1.0	1024	0.45684	0.34817
100	1.0	0 (fixed k-value)	1024	0.51443	0.32800
100	1.0	0.2	1024	0.52646	0.34388
100	1.0	0.3	1024	0.51529	0.31875
100	1.0	0.4	1024	0.54328	0.36120
100	1.0	0.5	1024	0.50599	0.32612
100	1.0	0.6	1024	0.54494	0.39655
100	1.0	0.7	1024	0.47917	0.30057
100	1.0	0.8	1024	0.49730	0.30880
100	1.0	0.9	1024	0.52598	0.36383
100	1.0	1.0	1024	0.49941	0.31733

## Data Availability

No new data were created or analyzed in this study. Data sharing is not applicable to this article as only publicly available datasets were used. These datasets (MNIST and CIFAR-10) can be found at: MNIST: http://yann.lecun.com/exdb/mnist/; CIFAR-10: https://www.cs.toronto.edu/~kriz/cifar.html, accessed on 8 December 2025.
